# *Escherichia coli* from Crohn’s disease patient displays virulence features of enteroinvasive (EIEC), enterohemorragic (EHEC), and enteroaggregative (EAEC) pathotypes

**DOI:** 10.1186/s13099-015-0050-8

**Published:** 2015-01-29

**Authors:** Ana Carolina da Silva Santos, Fernando Gomes Romeiro, Ligia Yukie Sassaki, Josias Rodrigues

**Affiliations:** Laboratory of Medical Bacteriology, Department of Microbiology and Immunology, Institute of Biosciences of the State University of São Paulo (UNESP), Brazil, Distrito de Rubião Junior, CEP 18618-970 Botucatu, SP Brazil; Department of Internal Medicine, Botucatu Medical School of the State University of São Paulo (UNESP), Brazil, Distrito de Rubião Junior, CEP 18618-970 Botucatu, SP Brazil

**Keywords:** *Escherichia coli*, Crohn’s disease, Invasive, MLST, Serotype, Adherence, Virulence

## Abstract

**Background:**

*Escherichia coli* is a normal inhabitant of the gut which upon acquiring virulence factors becomes potentially able to cause diseases. Although *E. coli* population augments in Crohn’s disease (CD), the reason of this proliferation is not yet clear. CD associated *E. coli* shows features of extraintestinal pathogenic categories (ExPEC), and eventually the ability to invade cultured epithelial cells, a property observed among diarrheagenic *E. coli* (DEC). In this work, data on the characterization of an *E. coli* isolate from a CD patient reveal that, besides invasiveness, CD associated *E. coli* may harbor other typical DEC markers, namely those defining enterohemorragic (EHEC) and enteroaggregative (EAEC) pathotypes.

**Results:**

The studied strain, detected both in an ileum biopsy and stools, belonged to the B2 *E. coli* reference collection (EcoR) phylogroup and harbored the intimin, Shiga cytotoxin 1, and AggR transcriptional activator encoding genes (eae, stx1, aggR, respectively); displayed aggregative adherence to Hep-2 cells and an ability to enter Caco-2 cells four times as high as that of EIEC reference strain and half of invasiveness of AIEC LF82. It was able to enter and replicate in J774 macrophages with invasiveness 85 times as high as that of LF82, but with only one sixth of the intracellular proliferation ability of the later. Extracellular products with cytotoxic activity on Vero cells were detected in strain’s cultures. Preliminary analysis indicated similarity of this strain’s genome with that of O104:H4/2011C-3493.

**Methods:**

Following its isolation from a resected CD patient, the strain was characterized by in vitro adhesion and invasion assays to Hep-2, invasion to Caco-2 cells and to J774 macrophages and tested for the ability to form biofilm and to produce Shiga cytotoxins. PCRs were carried out to identify virulence genetic markers and for EcoR phylogrouping. The strain’s genome was sequenced by means of Ion torrent PGM platform.

**Conclusion:**

The detection, in a CD patient, of an *E. coli* combining virulence features of multiple DEC pathotypes seems not only to stress the relevance of *E. coli* to CD etiopathogenesis but also to indicate the existence of new and potentially more virulent strains putatively associated with this disease.

## Background

Crohn’s disease (CD), one of the clinical variants of inflammatory bowel diseases (IBD), is characterized by chronic lesions of varying intensity along the gastrointestinal tract resulting from an exacerbated reaction of a defective immune system. The stimulus for the anomalous immune response is credited to the local resident microbiota which, in CD patients, presents with a reduced biodiversity along with the abundance of particular bacterial species such as *Escherichia coli*. The success of *E. coli* proliferation under this pathological condition has been explained by its capacity to metabolize nitrogen and sulfur by-products of inflammation, outcompeting anaerobes [1]. In addition, this elevation is particularly evident in the gut mucosa [2] where they are able to translocate M cells in Peyer patches and lymphoid follicles, which is the site of early lesions [3,4]. Differential characteristics of the augmented *E. coli* population include multi-drug resistance [5], association with B2 and D phylogroups [6] and the ability to interact with different kinds of epithelial cell lineages [7]. The later observation led to the description of adherent and invasive *E. coli* (AIEC), following extensive characterization studies on the LF82 strain [8]. In *in vitro* assays, AIEC is able to attach to and to invade non-intestinal and intestinal epithelial cells [7,9] and to survive inside macrophages [10]. In macrophages, the internalized bacteria not only keep the cell viability but also replicate and trigger the expression of TNF-α, a pro-inflammatory cytokine. Although these cell interaction strategies resemble those employed by enteric pathogens such as *Salmonella* and classical enteroinvasive *E. coli* (EIEC), AIEC do not share their virulence background [9]. The ability to persist inside macrophages has turned AIEC suspected of involvement in the formation of granulomas usually found in intestinal biopsies of CD patients [10]. Other AIEC strains with similar virulence properties to those of LF82 have been identified [11,12]. These strains are more similar to extraintestinal pathogenic (ExPEC) [13] than to classical diarrheagenic *E. coli* (DEC). We report here the results of characterization of an invasive *E. coli* detected in the ileum and stools of a CD patient, who underwent terminal ileum resection. Although this strain is able to attach to and invade epithelial cells, it does not fit all the criteria that define AIEC [14]. Differences in relation to AIEC include the possession of a classical enteropathogenic *E. coli* (EPEC) O antigen and features of enterohemorragic and enteroaggregative pathotypes.

## Results

### Biochemical profile, O:H serotype and genetic markers

**Figure 1:**
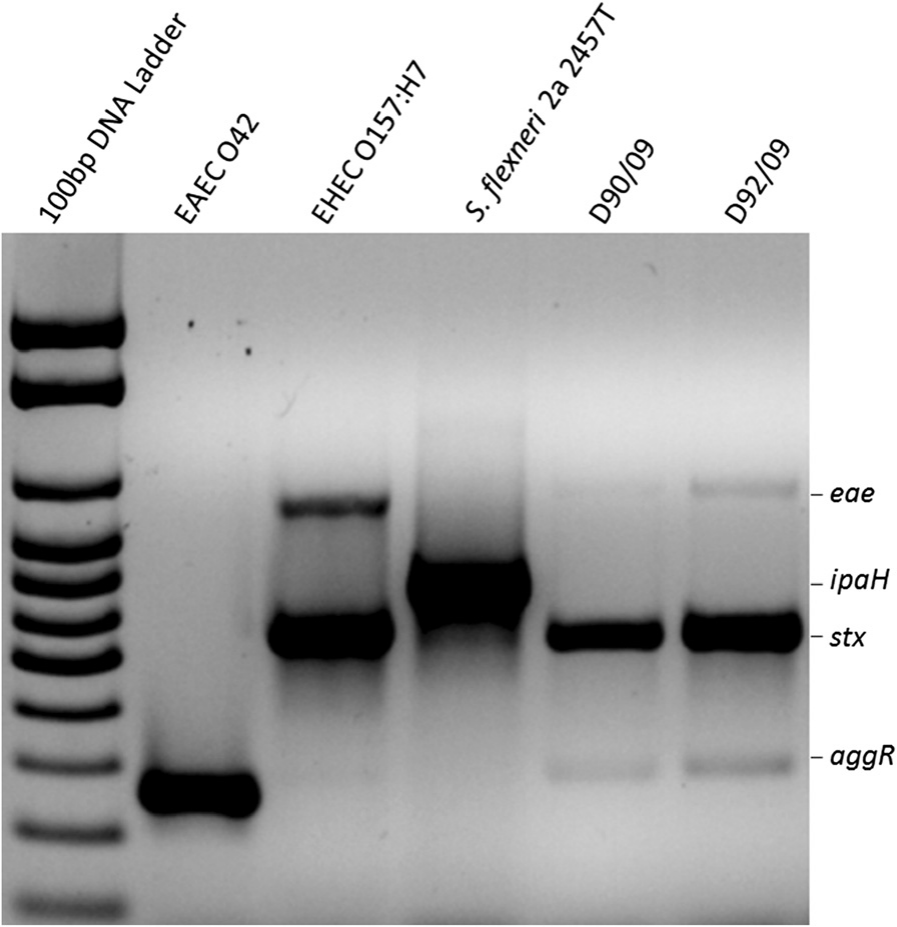
**Agarose gel electrophoresis showing PCR amplicons of virulence genetic markers detected in isolates D90/09 and D92/09 (5, 6) and positive control strains (2, EAEC O42** ***aggR;*** **3, EHEC** ***eae*** **and** ***stx;*** **4,** ***S. flexineri ipaH*** **)** ***.*** Lane 1, 100 bp DNA ladder.

All of 10 randomly chosen colonies from an ileum biopsy MacConkey agar culture and an equal proportion and number of colonies from a stools culture in this same medium were identified as *E. coli.* All 10 colonies from each clinical sample showed an identical biochemical profile (positive results for lysine decarboxylase, movement, glucose and lactose fermentation, gas from glucose fermentation and indole production; and negative results for urease, L-tryptophan deaminase, citrate utilization, and hydrogen sulfide production). Given the biochemical identity of all 20 colonies only an isolate (a single colony) from each clinical sample (D90/09 from stools and D92/09 from ileum) was selected for PCR screening, O:H typing, invasion assays, EcoR phylogroup and MLST classification. PCRs carried out to identify DEC typical genetic virulence markers and some serine protease autotransporters of *Enterobacteriaceae* (SPATE) genes revealed the presence of *eae, stx1, aggR, sat, espC, tsh* and *vat* in both isolates. Figure 1 shows DEC markers results of the multiplex PCR, according to Toma et al. [15]. The identification of the *stx* variant (*stx1*) was performed in a separate PCR (not shown). Specific PCR for EcoR phylogroup classification enabled the assignment of the isolates in the B2 phylogroup. O and H antigens were indirectly determined by manually searching sequences in the isolates’ genome (see below) matching genetic variants associated with the corresponding genes in GenBank. Thus, particular genome sequences of both isolates matched O126 gene cluster (DQ465248.1), and the H27 flagellin gene (AF345848.1) with 99% and 100% identity, respectively. The presence of O126 antigen in both isolates could be demonstrated by slide agglutination serological tests. Similarly multilocus sequencing typing (MLST) of D90/09 and D92/9 was based on the genome’s sequences, which upon being submitted to the Center for Genome Epidemiology website [16] was found to belong to ST3057 (Table 1).

**Table 1:** **Alleles profile and other parameters of MLST typing of the D92/09 as belonging to the 3057** ^**(a)**^

Locus*	**% identity**	**HSP length**	**Allele length**	**Gaps**	**Allele**
*adk*	100	536	536	0	290
*fumC*	100	469	469	0	54
*gyrB*	99,8	460	460	1	55
*icd*	100	518	518	0	324
*mdh*	100	452	452	0	35
*purA*	100	478	478	0	40
*recA*	99,7	381	510	1	223

^(a)^Output file created at the Center for Genome Epidemiology website [16].

*adk, adenylate kinase; *fumC*, fumarate hydratase; *gyrB*, DNA gyrase; *icd*, isocitrate/isopropylmalate dehydrogenase; *mdh*, malate dehydrogenase; *purA*, adenylosuccinate dehydrogenase; *recA*, ATP/GTP binding motif.

### Adherence and invasion

**Figure 2:**
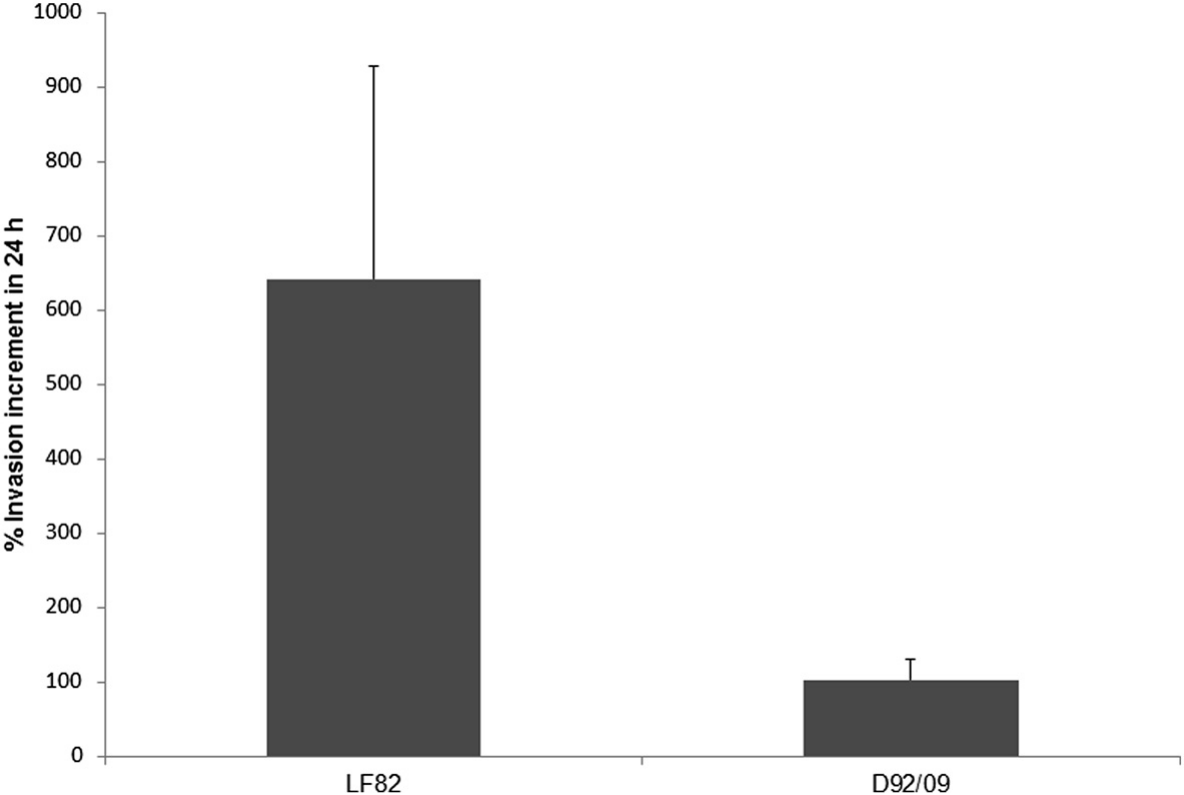
**Average percentage of LF82 and D92/09 invasiveness in macrophage line J774 within 24 h post-infection relative to the results obtained in a 3 h post-infection test.**

**Figure 3:**
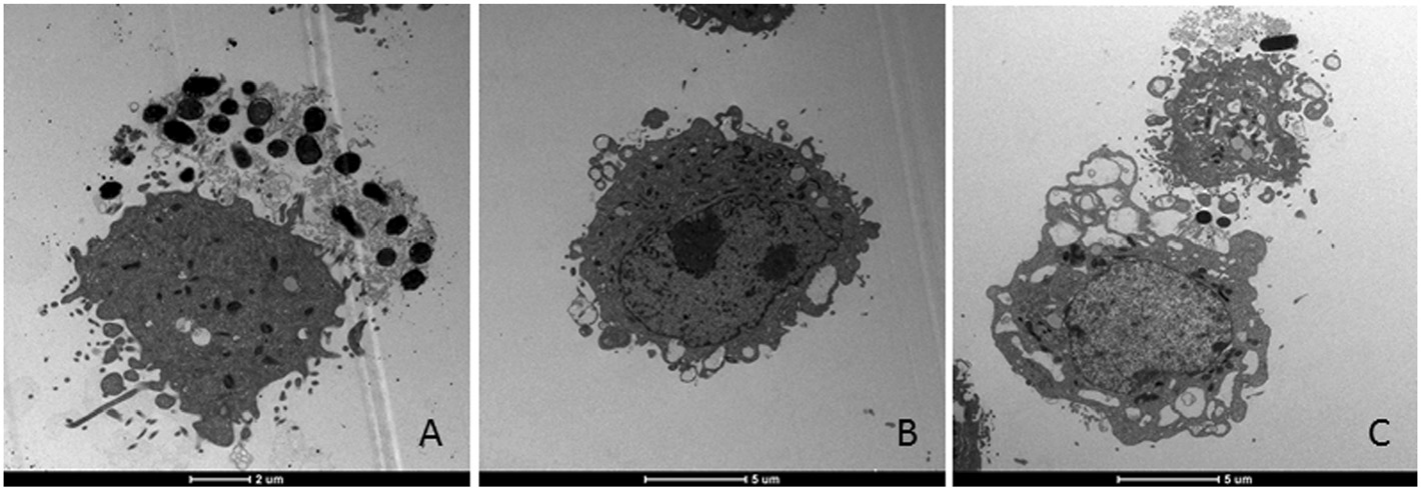
**Transmission electron microscopy of Hep-2 cells preparations infected with strain D92/09, where a profound cytotoxic effect both in parasitized (A) and in bacteria free cells (B and C) are observed.** In bacteria infected cell, an almost complete destruction leading to nuclear damage is seen **(A)**. In bacteria free cells cytotoxic effect manifested by membrane vesicles **(B)** and large cytoplasm vacuoles formation **(C)**.

All 20 isolates (10 from stools and 10 from ileum biopsies) were adherents to Hep-2, showing the characteristic “stacked brick” aggregative phenotype (AA). Given the phenotypic similarity of all *E. coli* isolates from the ileum and stools, and the phenotypic and genetic similarity between D90/09 and D92/09 they were all considered as a single clone, henceforth referred to as D92/09 strain. Thus only this isolate was submitted to the additional characterization assays. D92/09 was invasive to Hep-2 and Caco-2 cells, with an average percentage of internalized bacteria of 0.2% and 0.8%, respectively. The equivalent invasiveness values for the positive control EIEC strain used in the tests was 0.2% in both cell lineages. In the assays with Caco-2, LF82 was also included and displayed an invasion percentage of 1.7%. The average invasiveness of D92/09 in macrophage J774 was 35%, as compared to 0.41% of LF82. In the assays to evaluate intracellular replication, the number of internalized bacteria after a 24 h post-infection period was double it was within 1 h, a capacity of intracellular proliferation six times lower than that of LF82 (Figure 2). The intracellular localization of bacteria was inferred from the result of gentamicin sensitivity test, which indicated absence of bacterial viability in cultures with drug concentrations lower than10 μg/ml, 10 times inferior to that used to kill extracellular bacteria in the invasion assays. The invasive ability of D92/09 was confirmed by the observation of bacteria within Hep-2 cells in transmission electron microscopy (TEM) preparations (Figure 3A). The strain was found to be a weak biofilm former (mean SBF = 0.35 ± 0.5).

### Cytotoxic effect

**Figure 4:**
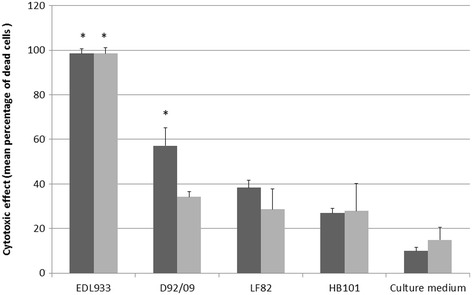
**Action of eight times diluted filtrates from bacterial culture supernatants (dark bars) and filtrates from polymyxin lysed bacteria suspensions (gray bars) on Vero cells.** Asterisks denotes statistically significant differences (*p* < 0.05) in comparison with LF82 in *χ*2 test.

Figure 4 shows the effects of incubation of eight times diluted D92/09 bacterial culture supernatants and lysates from polymyxin treated bacteria on Vero cells. In wells treated with these suspensions, an average 57% and 34% of supernatants and lysates respectively had dead cells. The cytotoxicity of D92/09 lysate preparation was not significantly different from that induced by both LF82 and HB101 lysates and supernatants. Yet, the higher percentage of dead cells in wells added with D92/09 supernatants indicated a more powerful effect of this preparation than those caused by suspensions (lysates or supernatants) from those of LF82 and HB101. Nonetheless, cytotoxic effect of D92/09 supernatants was lower than that of supernatant and lysates from EDL 933 (Figure 4). Although not aiming to detect a cytotoxic effect, TEM carried out to detect intracellular bacteria also revealed a strong cytotoxic effect induced by the strain (Figure 3). In TEM preparations, cytotoxicity manifested through the upsurge of cell membrane vesicles (Figure 3B) and the formation of cytoplasm vacuoles (Figure 3C), eventually affecting the nucleus (Figure 3A). The level of toxicity was more pronounced in bacteria parasitized cells where the cytoplasm appeared almost completely disintegrated and the nucleus severely damaged (Figure 3A).

### Preliminary genome analysis

The *E. coli* D92/09 DNA sequences were assembled in 341 contigs with an average size of 14,900 bp, totalizing 5,081,224 bp, 5842 coding sequence (CDS), 69 tRNAs and a GC content of 50,48%. A number of 145,211 bp from the whole genome corresponded to plasmid DNA, which showed 98% sequence identity with plasmid pO111_H2 (NC_013370.1). Chromosome sequences displayed 97% identity with O104:H4/2011C-3493 (NC_018658.1), a strain isolated from an American citizen diagnosed with hemolytic uremic syndrome (HUS) and who had traveled to Germany during the 2011 local HUS outbreak. Both plasmid and chromosome sequences included DNA from multiple Enterobaceriaceae bacteriophages, such as the *stx1* converting phage. The average GC content of these sequences deviated significantly from the corresponding value of the total genome. An additional marker of the D92/09 genome, observed in this initial analysis, was the *E. coli* long polar fimbriae (*lfpA-D*) operon. The strain’s sequence data have been submitted to the NCBI Sequence Read Archive under accession number SRP050886.

## Discussion

Although most *E. coli* lives as commensals in the gut, some strains can be associated with several human diseases ranging from mild acute diarrhea to fatal HUS and meningitis [17]. *E. coli* has also been suspected of involvement with IBD because of its numerical elevation in the gut of IBD patients [2,7,18], contributing significantly for dysbiosis in the local microbiota, which is viewed as an important factor associated with the cause or aggravation of the symptoms of the disease [19]. Most of characterization studies have indicated that potentially virulent *E. coli* strains from IBD usually do not bear typical virulence features of classical diarrheagenic pathotypes and frequently are phenotypic and genetically related to ExPEC [6,20]. This can be exemplified by AIEC LF82 which bears no DEC virulence genetic markers and belong to O83, a serogroup usually found in association with cases of urinary tract infections [21]. In addition to LF82, at least two other AIEC strains also of the serogroup O83 had its whole genome sequenced and the results revealed genetic similarity among them [13,22,23]. This work presents features of a CD *E. coli* (labeled D92/09), which although proved to be adherent and invasive to cultured epithelial cells, displayed many differences in relation to AIEC, regarding identification (typing) markers and virulence. It belongs to the O126:H27 serotype, to B2 EcoR phylogroup, which includes virulent strains usually possessing pathogenicity markers of UPEC [24] and to the ST3057, an uncommon clone first isolated from pig in Hong Kong in 2009 and considered as commensal [25]. Besides invasiveness, D92/09 harbors *eae* and *stx1* of enterohemorrhagic (EHEC) and *aggR* and aggregative adherence phenotype of enteroaggregative (EAEC) *E. coli*.

In many aspects, the interaction of D92/9 with cultured cells was also different from that of AIEC. For example, D92/9 uptake by macrophage J774 was 87.5 times as high as that of LF82 and the intracellular replication 24 h post-infection was only one sixth of that shown by LF82. The number of Hep-2 cells internalized D92/9 bacteria increased largely after 4 h post-infection (data not shown) and they proved to be not only invasive but also cytotoxic. Bacteria cytotoxicity could be noticed by cell detachment from bacteria infected Hep-2 cell monolayers observed at the light microscope in the adhesion assays (not shown) and by TEM, where bacteria infected and non-infected cells appeared damaged. These observations were confirmed by the results of Vero cell assays, where diluted bacterial culture supernatants caused cell death in levels significantly higher than that induced by culture extracts from LF82 or HB101. Since the strain had *stx1*, these effects might have resulted from the action of Stx1, along with other cytotoxins such as the SPATEs, whose genes it also carries. However, the percentage of dead cells in monolayers treated with extracts from D92/9 was lower than that of monolayers under the action of corresponding EDL 933 products. One possible explanation for this difference is that D92/9 lacks Stx2, which is expressed by EDL 933 along with Stx1. In experimental infections, Stx1(+) rabbit diarrheagenic *E. coli* (RDEC) has been shown to cause multiple injuries and more severe inflammation than Stx1(−) RDEC [26]. Stx cytotoxins are made up of a single A subunit and five B subunits. The A subunit cleaves an adenine residue of the rRNA 28S component of the target eukaryotic cell ribosome, blocking protein synthesis. The B subunits bind to globotriaosylceramide (Gb3), a membrane glycolipid which was found to be widely distributed among rabbit absorptive villus epithelial cells [27]. Several works have reported the isolation of Stx producing *E. coli* (STEC) in ulcerative colitis [28-30], but as far as we are concerned, possibly no reports on the isolation STEC from a resected CD patient has been previously published. On the other hand, D92/09 displayed some AIEC features, such as the presence of *lpfA-D* which has been shown to contribute for bacteria invasiveness and translocation across M cells [8]. As a strain belonging to B2 phylogroup, it also harbored *chuA,* a gene involved in heme acquisition and which has been correlated with bacterial persistence inside macrophages [31]. Serogroup O126 is very heterogeneous, including strains lacking known virulence markers [32], as well as potentially virulent strains of distinct pathotypes found both in humans and animals [33-35]. O126:H27 is a classical EPEC serotype previously associated with outbreaks [36] and sporadic cases of diarrhea [37]. The present work possibly is a rare report on the identification of this O:H type in CD.

In addition to *stx1*, the strain of the present study carried *eae* and *aggR.* The former encodes intimin which is responsible for ultrastructural lesions in the host cell membrane at the site of bacteria attachment. These alterations, known as attaching and effacing [38], are believed to be the main component of inflammatory reactions accompanying EHEC infections [27]*. aggR* is a transcriptional activator of multiple virulence factors in EAEC [17]. According to previous reports, EAEC are dominant in IBD patients [39,40] and some EAEC strains are able to form biofilm and to induce mucus secretion [41] and interleukin-8 expression [42] by intestinal epithelial cells. Additional virulence factors of EAEC include serine proteases auto transporters of *Enterobacteriaceae* (SPATE).

Preliminary genome analysis revealed a high genetic identity of D92/09 with a strain belonging to O104:H4 [43], serotype involved in the 2011 deadly HUS outbreak in Germany [44]. Phenotypic identity, such as the rare combination of characteristic from EAEC (AA adherence) and STEC (Stx1) was also observed. The connected action of these diverse pathogenicity factors was considered as the reason for the high virulence of the O104:H4 strains in the European HUS outbreak [44]. In addition to these traits, D92/09 proved to be invasive, a property common to pathogens associated with intestinal inflammation, such as *Salmonella* and *Shigella*. Some particularities of the intestinal microenvironment in CD patients, such as a more permeable mucosa and altered mucus production, turn it even more vulnerable to installation of pathogens such as the strain of this study. In fact, the bacteria were detected in an intestinal site where multiple erosions were observed. While this observation cannot imply a causal relationship, an eventual involvement of the bacteria cannot be ruled out. Both chromosome and plasmid DNA displayed a number of bacteriophages, including the *stx1* converting phage, indicating that at least some of the strain’s virulence properties were acquired by horizontal transfer. The plasmid DNA sequences were very similar to pO111_2, detected in an O111 EHEC and which was previously shown to share multiple virulence genetic determinants with EHEC of other serogroups [45].

Previous work has reported an elevation in the number of *E. coli* in postoperative recurrence of CD [46]. Eventually, the possession of a diversified virulence background like that of D92/09 would represent an adaptive advantage within the augmented bacterial population in this clinical condition. Additional information drawn from a complete genome analysis as well as epidemiological studies will give a broader understanding of the significance of the presence of this *E. coli* in the resected CD patient.

## Methods

### Patient

The studied *E. coli* was isolated in December 2009 from stools and an ileum biopsy of a 51 year old woman who attended the Endoscopy unit of the University Hospital of the Botucatu Medical School/UNESP-SP, Brazil, for routine colonoscopy. The patient was diagnosed about one year earlier with small bowel CD complicated by an obstructive stenosis adjacent to the ileocecal valve and subsequently submitted to a terminal ileum and cecum resection. The patient had no clinical symptoms and was not under antibiotics or other drug treatment for at least two months preceding the clinical specimen collection. Yet, at colonoscopy examination several mucosal erosions (ca. 1–2 mm in size) and fibrinous lesions 6 mm in size could be observed in the ileum near to the anastomosis site. There was no colonoscopy finding in the mucosa of the entire length of the colon as well as in the rectum. The biopsy analyzed in this study was sampled from the ileum lesions. The patient was informed on the purpose of this study and manifested her agreement by signing a consent form. This study was approved by the local Committee on Ethics in Research. CD diagnosis was based on conventional clinical, colonoscopy and histopathological criteria [47].

### Bacterial isolation

Unless otherwise indicated, all the culture media and reagents for bacterial growth were manufactured by Oxoid (Basingstoke, Hampshire, England) and the culture conditions were 37°C per 18-24 h. *E. coli* was identified among colonies grown from a freshly collected (within 3 h after colonoscopy) ileum biopsy and a 2 days refrigerated stools sample directly streaked on MacConkey agar plates. A number of 10 lac + colonies from these cultures were transferred to semi-solid MILi, modified Rugai [48] and Simmons citrate agars (BBL, Cockeysville, USA) wherein the following reactions could be detected: urease, indole, and H_2_S production, lactose and glucose fermentation, gas from glucose fermentation, sodium citrate utilization as single carbon source, bacterial movement, lysine decarboxylation and L-tryptophan deamination. Those colonies showing positive results for indole and glucose fermentation and negative results for H_2_S, L-tryptophan deamination and urease production were considered as *E. coli*. They were then incubated overnight in brain heart infusion broth and the resulting cultures mixed with 15% glycerol and stocked at −80°C.

### Adhesion assays

Bacterial adherence was tested in Hep-2 cells (ATCC CCL-23) monolayers grown to semi-confluence on glass coverslips put in 24 wells microplates (Kasvi, Curitiba, Brazil), filled with 0.7-1.0 ml minimal essential medium Eagle (MEME) (Sigma, St. Louis, USA), supplemented with 5% heat inactivated fetal calf serum (FCS) (Cultilab, Campinas, Brazil), streptomycin, penicillin (Sigma, St. Louis, USA) and amphotericin B (Sigma, St. Louis, USA). Unless otherwise indicated, in all steps below of adhesion and invasion assays, cells incubation was carried out at 37°C in normal (room) concentrations of CO_2_ for bacteria, and in 5% CO_2_ atmosphere for non-infected or bacteria infected epithelial cells.

After washing the monolayers thrice in phosphate buffered saline (PBS), 0.7-1.0 ml of fresh antibiotic free MEME/FCS containing 1% D-mannose (Sigma, St. Louis, USA) and 20 μl of overnight Luria Bertani (LB) bacterial cultures were added to each well and the plates incubated for 3 hours. Following 3 h incubation, the preparations were washed thrice in PBS, fixed in methanol (Labsinth, Diadema, Brazil) and stained in May-Grünwald and Giemsa (Sigma, St. Louis, USA). The coverslips were mounted on glass-slides and observed under light microscope for attached bacteria and adherence phenotype.

### Invasion assays

Hep-2 cells were cultured in 35 mm Ø Petri dishes to post confluent monolayers formed from an inoculum of 2×10^5^ cells/cm^2^ seeded 20 h before in MEME/FCS and grown under the same conditions described above. After the medium removal, the monolayers were washed thrice in PBS and infected with a number of bacterial cells 10 times as high as that of Hep-2 cells inoculum [multiplicity of infection (MOI) of 10]. The bacteria infected cells were incubated in antibiotic free MEME plus 10% FCS for three hours and washed four times after the removal of the medium. Fresh MEME plus 10% FCS and 100 μg/ml gentamicin (Sigma, St. Louis, USA), to kill extracellular bacteria, were then pipetted in the plates and the preparations were again incubated for one hour. Afterwards, the cells were washed thrice in PBS and lysed by incubation for 5 minutes at room temperature (RT) in 1% Triton X-100 (Sigma, St. Louis, USA). Cell lysates in volumes of 100 μl were spread on nutrient agar plates and incubated overnight. Internalized bacteria were quantitated by counting the number of colony forming units (CFU) grown on the plates. Bacterial invasion was expressed by the percentage of bacteria found in the lysates from the total inoculated on the cell monolayers. To exclude the possibility that the presence of bacteria in the lysates was due to gentamicin resistance, the strain was subjected to gentamicin minimal inhibitory concentration (MIC) determination by using Mic Evaluator strips (Oxoid, Basingstoke, England) on Mueller Hinton (Oxoid, Basingstoke, England) agar cultures, following the manufacturer’s recommendations. The invasion assays were carried out in triplicates and included AIEC LF82 and EIEC reference strain as positive controls and an *E. coli* K12 HB101, as negative control. Invasion assays in Caco-2 (CCIAL 063) was performed as described above, except that a longer incubation time (48 h) was necessary for cell growing to post-confluence, the concentration of CO_2_ in incubator was 10%, and the culture medium was Earle/199 (IAL, São Paulo, Brazil). The strain was also investigated for the ability to invade and replicate in the murine macrophage-like cell lineage J774 according to previously described procedures [10]. These cells monolayers were prepared by seeding and incubating for 18 h 10^5^ cells/cm^2^ in 35 mm Ø Petri dishes filled with RPMI 1640 (Sigma, St. Louis, USA) plus 10% FCS, penicillin, streptomycin and amphotericin B. For the invasion tests, the medium was removed from the monolayers, the preparations were washed thrice in PBS, and fresh antibiotic free RPMI 1640 plus 10% FCS was added to the plates. The monolayers were then infected with overnight LB bacterial cultures, at a MOI of 10, and incubated for 2 h. Afterwards, the medium was removed, the monolayers were washed thrice, new RPMI 1640 plus 10% FCS and 100 μg/ml gentamicin was added, and the preparations was incubated for 1 h. To determine the bacterial ability to enter the cells, the monolayers were lysed by RT incubation in 1% Triton X-100 for 5 minutes and 100 μl of lysates were subjected to overnight cultures in nutrient agar, for UFC counting. In tests to evaluate the bacterial capacity of intracellular replication, new RPMI 1640 plus 10% FCS and 20 μg/ml gentamicin were added to the plates after the monolayers washings. The preparations were then subjected to an additional 24 h incubation period, the monolayers were lysed and the lysates incubated as above, for intracellular bacteria counting. As mentioned elsewhere all invasion assays described above were carried out in triplicates, with individual replicates assayed in distinct days and the results expressed as the average percentage of internalized bacteria from the total added to the cells.

### Test for specific biofilm formation

The test to evaluate the strain ability to form biofilm followed the protocol of Martinez-Medina et al. [49], with some modifications. Bacteria were initially grown overnight in Luria-Bertani broth plus 5% glucose (LBG). Then 130 μl of LBG growth diluted 100 times in MEME/FCS plus 8% glucose (MEME/FCS/G) were dispensed in each well of 96 wells polystyrene microplates (Kasvi, Curitiba, Brazil). The plates were incubated at 30°C overnight and the optical density (OD) of the resulting culture measured at 620 nm. Afterwards, the cultures were removed, the wells were washed gently with PBS, and the plates dried. The preparations were stained with 1% Crystal violet solubilized in ethanol and the OD of the suspensions measured at 540 nm. EAEC strain 042 and bacteria-free MEME/FCS/G were respectively included as positive and negative controls in each test. Specific biofilm formation (SBF) was expressed by the formula: SBF = (A-B)/C, where A and B are the OD_540_ of the stained culture suspensions and the stained bacteria-free MEME/FCS/G suspensions respectively and C is the OD_620_ of the cultures in MEME/FCS/G. The studied strain and the controls were tested in 10x replicates. SBF values defined the ability of the strain to form biofilm as weak (≤0.5), moderate (0.5-1.0), or strong (≥1.0) [49].

### PCR screening

PCRs were used for screening the bacteria for virulence genes that distinguish the main diarrheagenic *E. coli* (DEC) pathotypes [38] and to detect genes whose combination enable the classification of the strains in one of the four major phylogroups (A, B1, B2 and D) of the *E. coli* reference collection (EcoR).

The search for DEC specific genetic markers was carried out by multiplex PCRs designed by Toma et al. [15]. DEC distinguishing genes were intimin encoding *eae* of EPEC and EHEC; *stx*, sequence common to shiga cytotoxins family genes of shiga toxin *E. coli* (STEC); *aggR,* gene for AggR, a regulator of multiple EAEC virulence genes; *elt,* heat labile toxin gene of enterotoxigenic *E. coli* (ETEC); and *ipaH,* plasmid borne IpaH gene of EIEC and *Shigella* sp. Negative control strain for the PCR was *E. coli* HB101, and positive controls were the following: EHEC EDL0933, for *eae* and *stx;* EAEC 042, for *aggR;* 40 T, for *elt*; and *Shigella flexneri* 2a 2457 T for *ipaH.* PCR for identification of *stx* variant (*stx1* or *stx2*) was done by multiplex PCR according to Paton et al. [50]. DNA template in each reaction mixtures were added as eluates of commercial purification kits (Qiagen, Valencia, USA) or crude cell lysates resulting from 10 minutes boiling of bacterial suspensions in sterilized water. The PCR cycling conditions, the primers (Eurofins, Huntsville, USA) concentrations and the remaining mix components were used according to the original reference protocol [15]. The *Taq* DNA polymerase, dNTP and other components of the master mix were used as ready commercial kits (NEB, Hitchin, UK). The template DNA was added to the mix components in a 0.2 ml polypropylene tube and the PCR reaction run in a MasterCycler ProS Thermocycler (Eppendorf, Hamburg, Germany). PCR amplicons were electrophoresed in 1% agarose gels containing 10^4^ x diluted Gel Red (Biotium, Hayward, USA) along with a100bp DNA size reference Marker (Norgen, Thorold, Canada). The electrophoresis separated DNA was observed in a gel doc EZ Imager (Biorad, Hercules, USA).

The PCR for assessment of EcoR phylogroups was based on the Clermont et al’s protocol [51], with modifications suggested by Doumith et al. [52]. The following DNA sequences were used as targets of amplification: *gadA,* which encodes glutamate decarboxylate-alpha, *chuA,* a gene involved with heme transport in *Shigella* and EHEC, *yjaA,* a sequence of unknown function and TSPE.C2, an anonymous DNA fragment. The primers employed for amplification of each sequence, the PCR reaction conditions and mixture components followed previously published work [52] and the PCR products were detected according to the description above.

### Assays in Vero cells

Evaluation of D92/09 cytotoxic effect was carried out on Vero cells (ATCC CCL-81), by testing supernatants and bacterial cell lysates from cultures grown on Penassay broth, as previously described [53]. EHEC strain EDL933 was used as positive control and *E. coli* K12 HB101 and bacteria-free culture medium were used as negative controls; for comparative purpose, AIEC strain LF82 was also tested. Bacterial cells lysates and culture supernatants were obtained as follows: 10 ml of overnight bacterial cultures were transferred to Erlenmeyer flasks containing 25 ml of fresh medium and incubated under shaking (150 rpm) for 18 h. The cultures were spun at 7000 x g for 10 min and the supernatants were filtered in 0.22 μm nitrocellulose membranes and aliquoted. The pellets were washed twice in PBS, re-suspended in 1 mg/ml of polymyxin B solution (Sigma St Louis, USA, cod. 81271) and incubated at 37°C for 30 min. The resulting suspensions were then centrifuged at 7000 × g for 10 min, the supernatants carefully removed and filtered in 0.22 μm nitrocellulose membranes.

The tests were performed with Vero cells incubated for 24 h, from an inoculum of 1,5×10^5^ cells per well of a 96 wells polystyrene microplate (Kasvi, Curitiba, Brazil). Culture medium was removed from the plate, the cells were washed three times in PBS, and 150 μl of doubling serial dilutions in Earle 199 medium (IAL, São Paulo, Brazil) of bacterial lysates filtrates or culture supernatants filtrates were added to each well. The plates were incubated at 37°C under 5% CO_2_ atmosphere and the cells observed within 24, 48 and 72 h. The cytotoxic effect was expressed as the average percentage of dead cells per well.

### Genome sequencing

D92/09 strain whole genome has been sequenced using the Ion torrent personal genome machine (PGM) (Life Technologies, Guilford, CT) with a 314 chip and 400 bp sequencing chemistry. DNA purification was performed from an overnight bacterial culture in LB using the QIAmp mini kit (Qiagen, Hilden Germany). The library was prepared using 1 μg of DNA and an Ion Xpress Plus fragment library kit comprising the Ion Shear chemistry according to the user guide. The resulting DNA fragments were separated in an E-gel electrophoresis system (Life Technologies, Guilford, CT) from which 400 bp fragments were recovered. The fragments were then ligated to ion sphere particles (ISP) for clonal amplification in an emulsion PCR in the Ion OneTouch instrument (Life Technologies, Guilford, CT). After checking the quality of amplification, the ISP beads were loaded onto the 314 chip, where the sequencing proceeded in the PGM. *De novo* genome sequences assembly was carried out with the Assembler plugin using the *E. coli* strain LF82 genome as a reference. Sequence features and sequencing quality parameters were accessed by the Torrent Browser software, where the files required for sequence analyses were remotely read in the Torrent Server. The following tools were used for the data analyses: Artemis software [54], which determined the coding sequences (CDS), GC content and other genome features, and tRNA scan [55]. For the multilocus sequence typing, a fastaQ file was submitted to the Center for Genome Epidemiology website [16], which generated a file with the sequences of the housekeeping genes (*adk,* adenylate kinase; *fumC*, fumarate hydratase; *gyrB*, DNA gyrase; *icd*, isocitrate/isopropyl malate dehydrogenase; *mdh*, malate dehydrogenase; *purA*, adenylosuccinate dehydrogenase; *recA*, ATP/GTP binding motif) and the corresponding MLST. D90/09 genomic DNA was also sequenced, but it was done in a 314 chip together with DNA from 3 other strains (each identified by a particular barcode and none related to this study). The purpose of this strain sequencing was only to identify some distinguishing features of the strains. Thus the low number of reads enabled the sequencing of only part of the D90/09 genome but with good sequence quality and quantity sufficient for MLST and O:H type identification.

### Transmission electron microscopy

A Hep-2 cells monolayer prepared as described above in a 75 cm^2^ polystyrene flask microplates (Kasvi, Curitiba, Brazil) was inoculated with the strain D92/09 at a MOI of 10 and incubated for 5 h. The preparation was washed in PBS, treated with gentamicin (100 μg/ml in MEME/FCS) and incubated for 1 h. After an extensive washing in PBS, the bacteria infected cells were removed from the flask and centrifuged at 400 g. The supernatant was discarded and the cell pellet fixed in Karnovsky’s glutaraldehyde solution for 12 h, washed thrice in phosphate buffer 0.1 M pH 7.3 (PB) and post-fixed in 1% OsO4 in PB, for 2 h. The preparation was washed in distilled water and immersed in uranyl acetate 0.5% for 2 h. After dehydration in a graded series of acetone, the pellet was immersed in a 1:1 araldite-acetone mixture for 12 h at room temperature and embedded in epoxy resin. Ultrathin sections from the resin blocs were cut and contrasted with uranyl acetate and lead citrate, for the observation at the microscope.
